# Cosuppression of Sprouty and Sprouty-Related Negative Regulators of FGF Signalling in Prostate Cancer: A Working Hypothesis

**DOI:** 10.1155/2015/827462

**Published:** 2015-05-17

**Authors:** Stephen J. Assinder, Daniella Beniamen, Frank J. Lovicu

**Affiliations:** ^1^Disciplines of Physiology, School of Medical Sciences and Bosch Institute, University of Sydney, Sydney, NSW 2006, Australia; ^2^Anatomy and Histology, School of Medical Sciences and Bosch Institute, University of Sydney, Sydney, NSW 2006, Australia

## Abstract

Deregulation of FGF receptor tyrosine kinase (RTK) signalling is common in prostate cancer. Normally, to moderate RTK signalling, induction of Sprouty (SPRY) and Sprouty-related (SPRED) antagonists occurs. Whilst decreased SPRY and SPRED has been described in some cancers, their role in prostate cancer is poorly understood. Therefore, we hypothesise that due to the need for tight regulation of RTK signalling, SPRY and SPRED negative regulators provide a degree of redundancy which ensures that a suppression of one or more family member does not lead to disease. Contrary to this, our analyses of prostates from 24-week-old* Spry1- *or* Spry2-*deficientmice, either* hemizygous *(+/−) or* homozygous *(−/−) for the null allele, revealed a significantly greater incidence of PIN compared to wild-type littermates. We further investigated redundancy of negative regulators in the clinical setting in a preliminary analysis of Gene Expression Omnibus and Oncomine human prostate cancer datasets. Consistent with our hypothesis, in two datasets analysed a significant cosuppression of SPRYs and SPREDs is evident. These findings demonstrate the importance of negative regulators of receptor tyrosine signalling, such as Spry, in the clinical setting, and highlight their importance for future pharmacopeia.

## 1. Introduction

Worldwide, prostate cancer accounts for one death every 4 minutes. It is the most commonly diagnosed cancer and the second leading cause of cancer death in men. The economic impact of prostate cancer is substantial. In 2010, prostate cancer is estimated to have cost AU$204,136,795 in Australia alone [1]. With estimated increases in the elderly population and increased survival rates [1], the burden of this disease will escalate significantly. The limited treatment options available result in significant morbidity to the individual. Side effects include lost libido, impotence, and incontinence. Most cases of advanced prostate cancer become resistant to treatment and inevitably result in death. In their analysis of the economic burden of prostate cancer, Roehrborn and Black [1] conclude that “Costs of prostate cancer treatment are only likely to increase in the future unless new strategies are devised to reduce the number of diagnoses and/or focus treatment where it is clinically most appropriate.” There is an urgent need for (i) better treatments of prostate cancer; (ii) prognostic markers that inform patient care; and (iii) individualised therapies. Essential to the discovery of novel pharmacological agents for individualised cancer therapy is an understanding of how disruption of intracellular signalling pathways leads to the formation of cancer.

Hyperactivation of FGF signalling is evident in 80% of prostate cancers [2]. Several mechanisms result in hyperactivation, including increased FGF expression that correlates with increased Gleason score [3], increased FGF availability from extracellular matrix [4], and sensitisation to FGF due to increased receptor levels [4, 5]. Indeed, in a prostate epithelium-specific FGFR1 knock-in mouse model, activation of expression results in adenocarcinoma [6], whilst in the clinical setting, a single nucleotide polymorphism in the* FGFR4* gene is associated with poor prognosis of prostate cancer [7, 8].

**Figure 1:**
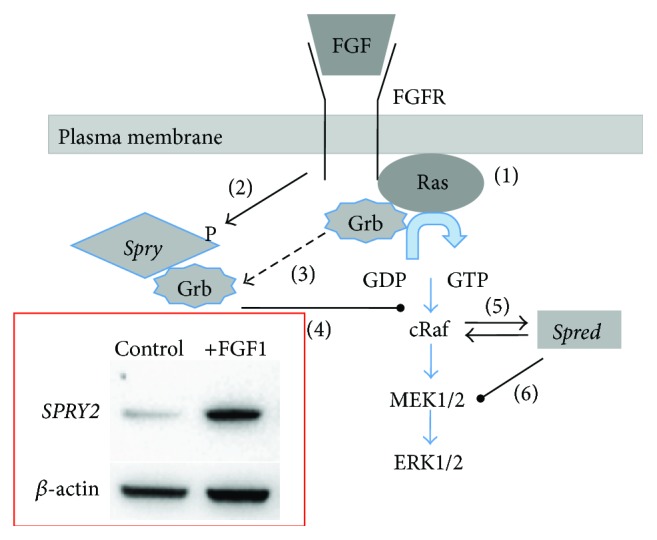
A stepwise overview of the mechanisms of sprouty and SPRED negative regulation of FGF signalling. Activation of the tyrosine kinase receptor (FGFR) results in (1) phosphorylation of Ras and subsequent activation of the MAPK signalling cascade (blue arrows); (2) FGFR activation also results in the direct activation by of Sprys by phosphorylation and increased expression via Ras-MAPK pathway (see inset: western blot of increased SPRY2 following FGF1 (10 ng·mL^−1^) treatment of normal prostate epithelial cells); subsequent sequestration of the FGFR signalling molecule Grb (3) by pSPRY results in (4) suppression of FGF signalling. Similarly, activation of FGFR results in Spred heterodimerisation and subsequent complexing with Raf (5) resulting in inhibition of MEK activation (6).

Normally, increased FGF signalling is counteracted by feedback inhibitors. Sprouty was one of the first negative feedback regulators of the FGF pathway to be identified, initially shown to be important for regulation of FGF-induced tracheal branching in* Drosophila* [9, 10]. Mammalian Sproutys are expressed in a highly restricted pattern that correlate with FGF signalling [11]. Spry is recognised in many physiological and developmental processes as an antagonist of receptor tyrosine kinase (RTK) signalling. Its overexpression mimics the functional loss of RTKs, including those activated by FGF [12, 13]. Overexpression of* Spry* in the developing chick limb bud inhibits cell differentiation, displaying a comparable phenotype to that reported in FGF null mutants [14]. Consistent with this, transfected cells overexpressing* Spry* have a reduced responsiveness to growth factors [15]. The exact nature of the inhibitory activity of Spry is unclear. Specific functions are exerted through multiple mechanisms, dependent on the growth factor stimulation and/or cell type [16]. For example, Spry can function as a decoy site, binding intracellular docking proteins, preventing the activation of intracellular signalling molecules, such as the MAPK/ERK1/2 pathway [17, 18]. Spry is selective for ERK1/2 signalling, with members exhibiting slightly different activities as they interact with different signalling proteins [18]. Each Spry protein has a conserved tyrosine residue (Tyr55/*Spry2*, Tyr53/*Spry1* and* Spry4*) that functions as a binding site for the SH2 domain of Grb2 [15]. In the case of FGF signalling, phosphorylated Tyr55 of Spry2 associates with Grb2, blocking the interaction of Grb2 with the FGF receptor adaptor molecule, FRS2, which bridges the FGF receptor to the ERK/MAPK pathway [18]. Hence, Spry can uncouple FGF-induced signal transduction leading to a block in ERK1/2 activation (Figure 1).

Spreds are also negative regulators of ERK/MAPK activation. Spred proteins primarily consist of three domains that (i) bind proline-rich sequences targeting Spreds to specific cellular sites where they function; (ii) allow tyrosine kinase interaction; and (iii) interact with cRaf to suppress ERK phosphorylation and negate FGF signalling (Figure 1).

Loss of SPRY has been reported in breast [19], liver [20], and lung [21] cancers. Functional studies have shown that suppression of SPRYs promotes a malignant phenotype in an* in vitro* model of breast cancer [19]. Direct injection of a dominant negative SPRY2 into mouse livers, with overexpression of *β*-catenin, induced neoplastic transformation [22]. Ectopic expression of* SPRY2* in cell lines derived from non-small cell lung carcinoma tissues significantly reduced proliferation and tumour formation of subsequent xenografts [21]. Lung tumourigenesis is unable to be induced by the carcinogen urethane in* SPRY2* overexpressing transgenic mice [23]. Similarly, loss of SPREDs in cancer is also evident. In hepatocellular carcinoma, both* SPRED1* and* 2* are downregulated, with an associated increase in invasion and metastasis [15, 24].

The role of SPRYs and SPREDs in prostate cancer is, however, poorly defined. There are limited reports of SPRY1 and SPRY2 suppression in clinical samples of prostate cancer [25, 26]. In support of a role for Sprouty as a tumour suppressor, proliferation of prostate cancer cell lines (LNCaP and PC3) is suppressed by* SPRY1* overexpression [25]. Recently, it was demonstrated that concomitant prostate-specific deletion of* Spry1 *and* Spry2 *in mice resulted in prostatic intraepithelial neoplasia (PIN), while deletion of either* Spry 1 *or* Spry 2* in hemizygous* Pten *null mice resulted in invasive carcinoma [27]. Only one report exists with regard to SPREDs in prostate cancer, describing evidence for a loss of* SPRED2* expression in high Gleason grade lesions [28]. Given this, we hypothesise that, due to the need for tight regulation of receptor tyrosine kinase signalling, having a family of SPRY and SPRED negative regulators provides a degree of redundancy where loss of one family member is not significant to disease formation. Until now, this has not been considered in the context of prostate cancer. Hence, in this study, we aimed to determine whether deletion of either* Spry 1 or Spry 2* alone could induce neoplastic changes in the mouse prostate, whilst also assessing public gene expression datasets to test the hypothesis that cosuppression of SPRYs and SPREDs is associated with aggressive prostate cancers. 

## 2. Material and Methods

### 2.1. Animals and Tissues

This study was approved by the University of Sydney Animal Ethics Committee under protocol number K03/5-2012/3/5763 and the tissue sharing scheme. Inbred male mice with germline deletions of either* Spry1 *[29] or* Spry2* [30] were housed under controlled temperature and 12 hr light/dark regime with food and water provided* ad libitum*. Mice with either homozygous allelic deletions of* Spry1 *(*Spry1* ^−/−^; *n* = 5) or* Spry2 *(*Spry2* ^−/−^; *n* = 2) or hemizygous allelic deletions of* Spry1 *(*Spry1* ^+/−^; *n* = 5) or* Spry2 *(*Spry2* ^+/−^; *n* = 5) and their wild-type (WT; *n* = 5) littermates were euthanized at 24 weeks postpartum by CO_2_ asphyxiation. Ventral prostates were removed and fixed in neutral-buffered formalin (NBF: 25 mmol·L^−1^ NaH_2_PO_4_; 50 mmol·L^−1^ Na_2_HPO_4_; 4% (w/v) formaldehyde). Following fixation, tissue samples were dehydrated and embedded in paraffin wax.

### 2.2. Histological Examination

Five *μ*m thin sections were cut and stained with haematoxylin and eosin. Stained tissue sections were observed by bright field microscopy by an observer blinded to the genotype. Tissue sections were assessed for normal acinar architecture and pathologies of low grade prostatic intraepithelial neoplasia (LGPIN) and high grade prostatic intraepithelial neoplasia (HGPIN) according to the Bar Harbor Classification of Mouse Prostate Pathologies [31]. At least 200 acini were scored for wild type, hemizygous, and homozygous prostates. The incidence of normal, LGPIN, and HGPIN acini was determined as a percentage of the total number of acini scored for each genotype and differences were determined by *R* × *C* test of independence and* post hoc* Pearson chi-square test.

### 2.3. Determination of Proliferative Index

Five *μ*m thin sections were assayed for immunoreactive proliferative cell nuclear antigen (PCNA) as a marker of proliferating prostatic epithelium. Briefly, sections were dewaxed in HistoChoice (Sigma-Aldrich) and rehydrated through graded alcohol before washing in phosphate buffered saline (PBS; pH 7.4) 3 times for 5 min each. High temperature antigen retrieval was then performed by immersion in preheated citrate/Tween-20 buffer (10 mMol·L^−1^ Na_3_C_6_H_5_O_7_; 0.05% (v/v) Tween-20; pH 6.0) and microwaving twice for 5 min at high power (600 W). Sections were left to cool for 35 min before washing 3 times for 5 mins each in PBS. A PCNA staining kit (Zymed Laboratories, Inc., South San Francisco, CA) was then employed according to manufacturers' instructions. In negative controls, the primary monoclonal anti-mouse PCNA antibody was replaced with 10% (v/v) normal mouse serum (Sigma-Aldrich, St Louis, MO, USA) or PBS (no antibody). As a positive control, 5 *μ*m thin sections of mouse testes were included. Following chromogen formation, sections were counterstained with haematoxylin, dehydrated, and cover-slipped with dibutyl phthalate xylene. Sections were viewed by bright field microscopy at high magnification under oil immersion. The number of total and immunopositive nuclei was counted in at least 4 fields of view for each animal by an observer blinded to the genotype. Proliferative index was determined as the proportion of PCNA-positive nuclei and a mean index determined. Any significant differences between mean proliferative indices for each of the* Spry1* and* Spry2 *genotypes were determined by one-way ANOVA and Tukey's HSD* post hoc* test.

### 2.4. *SPRY* and* SPRED* Gene Expression Analysis of Human Prostate Cancer cDNA Libraries

Two separate gene expression datasets lodged at the Gene Expression Omnibus, NCBI gene expression and hybridisation array data repository (http://www.ncbi.nlm.nih.gov/geo/), and on the Oncomine database (http://www.oncomine.org/), were assessed for* SPRY1, SPRY2, SPRED1*,* and SPRED2* expression. The GEO dataset (GDS1439: [32]) compares samples of benign prostatic hyperplasia (BPH) tissue with clinically localised primary prostate cancer tissue and with metastatic prostate cancer. The Oncomine dataset (Vanaja_Prostate; [33]) compares normal prostate with clinically localised primary prostate cancer tissue and with metastatic prostate cancer. Each gene was analysed for relative expression according to database output score. Coexpression was compared according to pathology and sum of ranks, where each gene's expression was assigned a rank score (where greater rank score indicates greater expression). Ranks of all 4 genes for each sample were summed and the sum of ranks was analysed for association by rank correlation.

## 3. Results

### 3.1. Single Germline Deletions of Either* Spry1* Or* Spry2* Result in PIN

**Figure 2:**
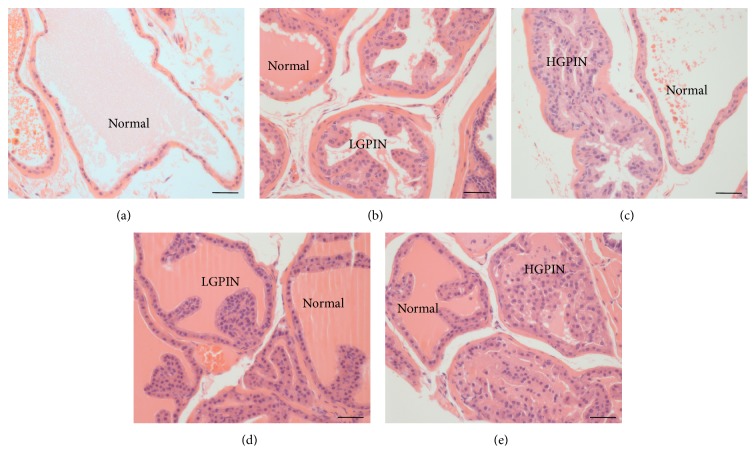
Histology of prostates from 24-week-old wild-type (a), hemizygous (b, c), and homozygous (d, e) null* Spry1* mice. Five *μ*m thin sections were stained with haematoxylin and eosin and assessed for normal acinar architecture and pathologies of low grade prostatic intraepithelial neoplasia (LGPIN) and high grade prostatic intraepithelial neoplasia (HGPIN) according to the Bar Harbor Classification of Mouse Prostate Pathologies [31]. Scale bar = 50 *μ*m.

Histological analysis of prostates from* Spry1* ^+/−^ or* Spry1* ^−/−^ mice determined the presence of normal acini, as well as acini displaying pathologies consistent with LGPIN and HGPIN (Figure 2). All* Spry1* ^+/−^ mice assayed had PIN pathology, whilst four of the 5* Spry1* ^−/−^ mice were determined as having PIN. No pathology other than ductal hyperplasia was apparent in wild-type mice prostates. The incidence of PIN pathologies, expressed as a percentage of all acini scored, was 29% for both genotypes and significantly greater than in wild-type littermates (*P* < 0.0001). Indeed, both* Spry1* ^−/−^ (14%; *P* < 0.0001) and* Spry1* ^−/−^ (25%; *P* < 0.0001) had significantly greater incidence of LGPIN than wild-type mice, where* Spry1* ^−/−^ prostates displayed significantly greater occurrence than* Spry1* ^−/−^ (*P* < 0.01). The incidence of HGPIN (15%) was significantly greater in the prostates of* Spry1* ^−/−^ mice than in either wild-type (*P* < 0.0001) or* Spry1* ^−/−^ (*P* < 0.05) mice. Whilst 5% of* Spry1* ^−/−^ acini were determined to have HGPIN, this was not significant when compared with wild type.

**Figure 3:**
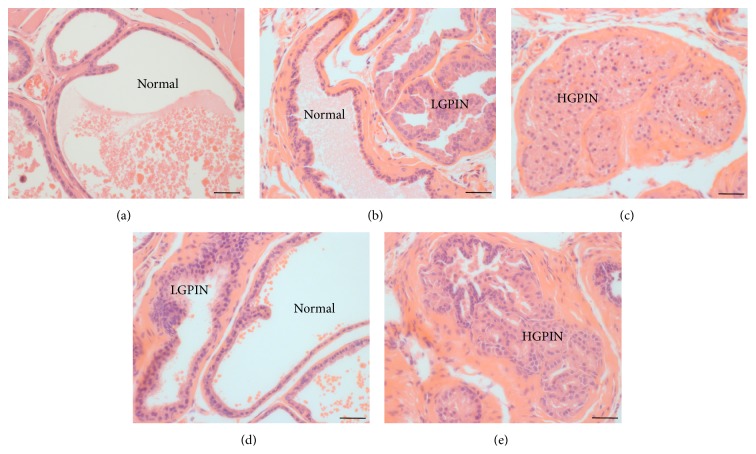
Histology of prostates from 24-week-old wild-type (a), hemizygous (b, c), and homozygous (d, e) null* Spry2* mice. Five *μ*m thin sections were stained with haematoxylin and eosin and assessed for normal acinar architecture and pathologies of low grade prostatic intraepithelial neoplasia (LGPIN) and high grade prostatic intraepithelial neoplasia (HGPIN) according to the Bar Harbor Classification of Mouse Prostate Pathologies [31]. Scale bar = 50 *μ*m.

Similarly, all prostates from* Spry2* ^+/−^ and* Spry2* ^−/−^ mice displayed pathologies of normal, LGPIN, and HGPIN (Figure 3). The sum of the incidences for these pathologies, expressed as a percentage of all acini scored, was 33% and 46% for* Spry2* ^+/−^ and* Spry2* ^−/−^, respectively, significantly greater (*P* < 0.001) than for prostates of wild-type mice that did not exhibit PIN. There was a significantly greater proportion of acini with LGPIN in both* Spry2* ^+/−^ (26%; *P* < 0.0001) and* Spry2* ^−/−^ (21%; *P* < 0.0001) prostates than in wild-type mice. Whilst the incidence of high grade PIN in the prostates of* Spry2* ^−/−^ mice (25%) was significantly greater than in* Spry2* ^+/−^ mice (*P* < 0.01) and wild-type mice (*P* < 0.0001), the incidence of HGPIN in* Spry2* ^+/−^ (7%) was not significant when compared with wild type.

### 3.2. Single Germline Deletions of Either* Spry1* Or* Spry2* Increase Prostatic Epithelial Cell Proliferation

**Figure 4:**
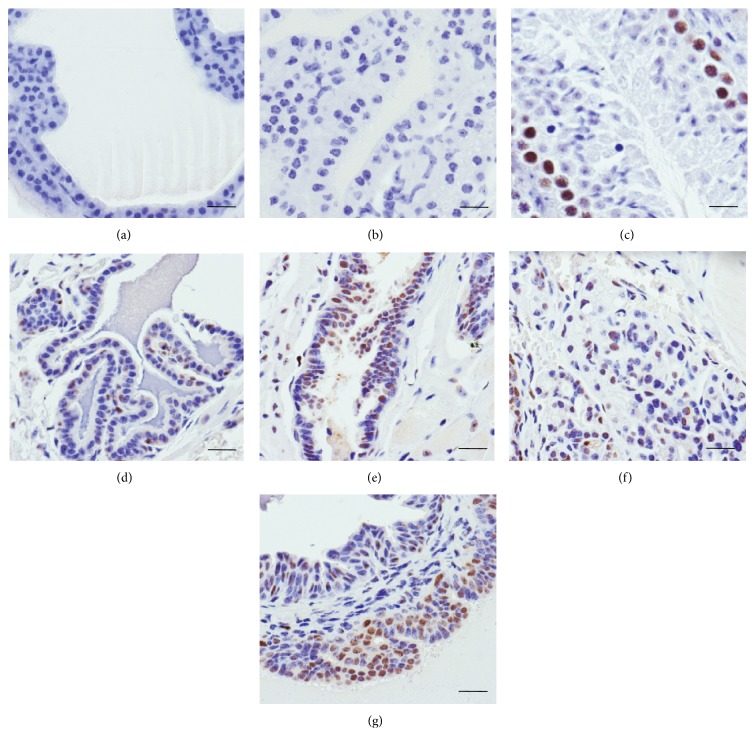
Immunocytochemical analysis of proliferating cell nuclear antigen (PCNA) protein in prostates of hemizygous and homozygous null* Spry *mice. Representative 5 *μ*m thin sections of (a) prostate, whole serum negative control; (b) prostate, no primary antibody negative control; (c) positive control of mouse testis showing immunopositive (brown) proliferating spermatogonia but immunonegative haematoxylin counterstained (blue) developing spermatids; immunopositive nuclei in (d)* Spry1* ^+/−^, (e)* Spry1* ^−/−^, (f)* Spry2* ^+/−^, and (g)* Spry2* ^−/−^ prostates. Scale bar = 25 *μ*m.

Consistent with an increased incidence of PIN pathologies in* Spry1 and Spry2 *hemizygous and homozygous mice was an increase in the number of PCNA-immunopositive ductal epithelial cells in the prostates of these mice. Whilst all animals exhibited proliferating cells, as determined by the presence of PCNA immunopositive nuclei (Figure 4), there were significantly (*P* < 0.001) greater proportions of immunopositive prostatic epithelia in* Spry1* ^+/−^ (26 ± 3%);* Spry1* ^−/−^ (31 ± 2%);* Spry2* ^+/−^ (16 ± 2%); and* Spry2* ^−/−^ (39 ± 3%) when compared with wild type (3.1 ± 0.5%). There was, however, no significant difference between the proportions of PCNA-immunopositive nuclei of* Spry1* ^+/−^ and* Spry1* ^−/−^ prostate epithelium. In contrast,* Spry2* ^−/−^ mice prostates had a significantly (*P* < 0.001) greater number of PCNA-immunopositive epithelia than* Spry2* ^+/−^ prostates.

### 3.3. Cosuppression of* SPRY* and* SPRED* Gene Expression Occurs in Human Prostate Cancers

**Figure 5:**
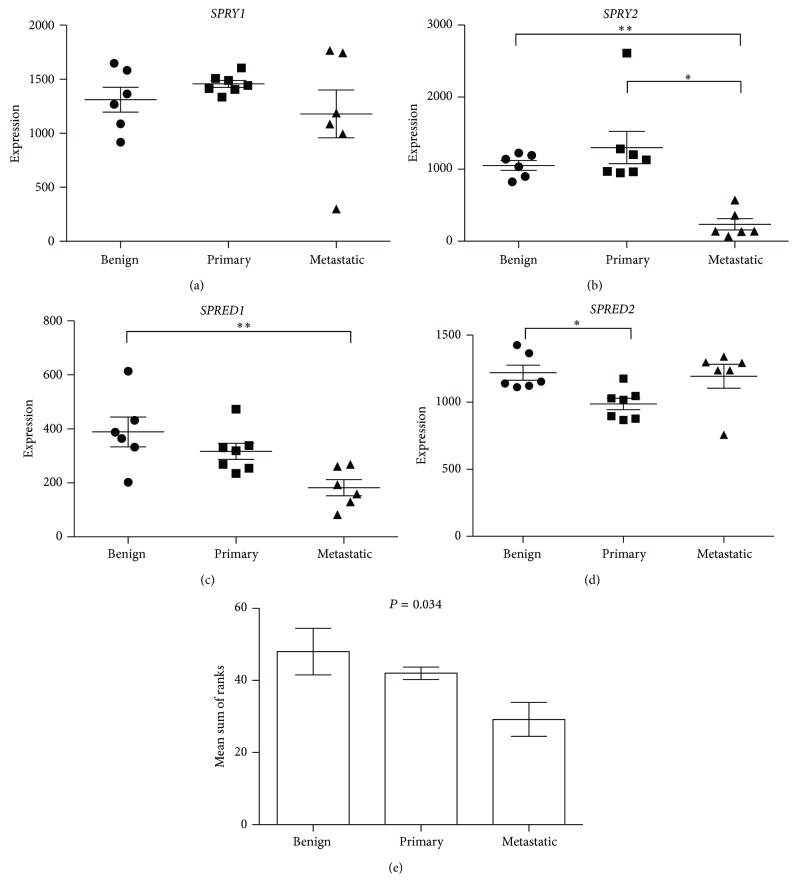
Analysis of (a)* SPRY1*, (b)* SPRY2*, (c)* SPRED1*, and (d)* SPRED2 *gene expression in Affymetrix gene chip hybridisation of mRNA (GEO dataset GDS1439; [33]) from benign prostatic hyperplasia (benign, *n* = 6), primary prostate carcinoma tissue (primary, *n* = 7), and metastatic prostate cancer (metastatic, *n* = 6). Individual data points are given with means (±sem) indicated by bar and whiskers of the MAS5-calculated signal intensity where the greater intensity indicates greater expression. Significant differences between means determined by one way ANOVA and Tukey's HSD* post hoc* test, where ^∗^ *P* < 0.05 and ^∗∗^ *P* < 0.01. (e) Analysis of mean (±sem) sums of each gene rank score for individual samples, according to pathology, for association by rank correlation.

**Figure 6:**
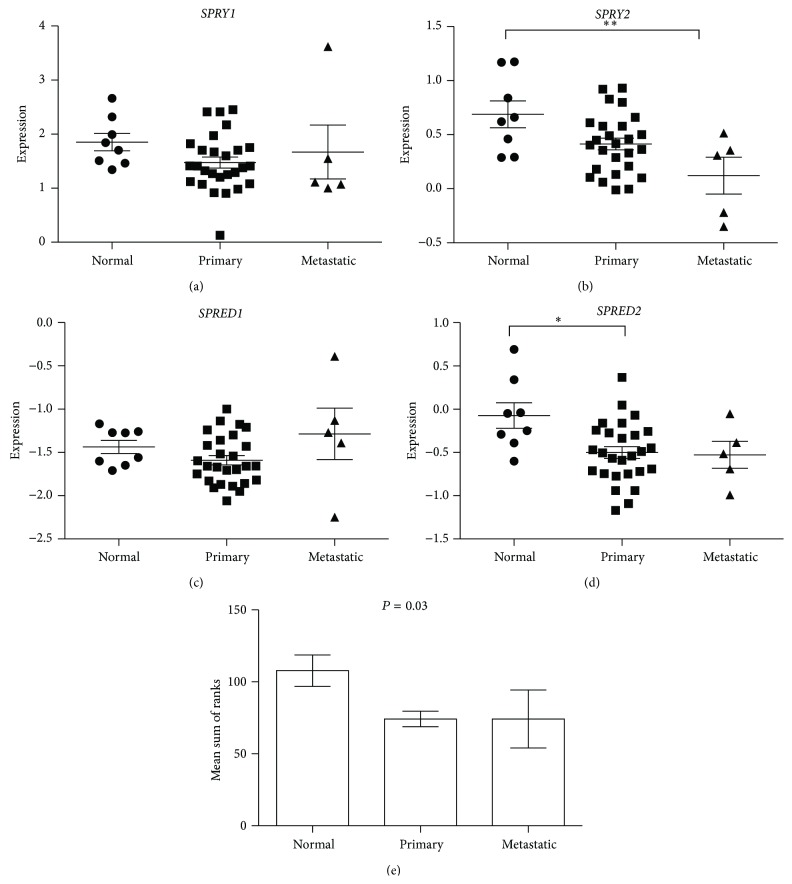
Analysis of (a)* SPRY1*, (b)* SPRY2*, (c)* SPRED1*, and (d)* SPRED2* gene expression in Oncomine dataset Vanaja_Prostate [34] from normal human prostate tissue (normal, *n* = 8), primary prostate carcinoma tissue (primary, *n* = 27), and metastatic prostate cancer (metastatic, *n* = 5) where individual data points are given with means (±sem) indicated by bar and whiskers of the log⁡2 median-centered signal intensity where the greater intensity indicates greater expression. Significant differences between means determined by one way ANOVA and Tukey's HSD* post hoc* test, where ^∗^ *P* < 0.05 and ^∗∗^ *P* < 0.01. (e) Analysis of mean (±sem) sums of each gene rank score for individual samples, according to pathology, for association by rank correlation.

No significant differences in* SPRY1* expression between noncancerous (benign prostatic hyperplasia, Figure 5(a); normal, Figure 6(a)) and prostate cancer tissues were evident. In contrast, both datasets displayed significantly decreased* SPRY2 *expression, with metastatic tissues having significantly suppressed expression compared with benign and primary carcinoma (Figure 5(b)) and with normal tissue (Figure 6(b)), respectively.* SPRED1 *expression was significantly reduced in metastatic prostate cancers in the Varambally Gene Expression Omnibus dataset when compared with benign tissue (Figure 5(c)) but no significant differences in* SPRED1* expression were evident between any tissue sites of the Vanaja dataset (Figure 6(c)).* SPRED2* was significantly decreased in primary prostate carcinomas in both datasets (Figures 5(d) and 6(d)). Analysis of coexpression by comparing the sum of rank scores of each gene for each individual sample in a dataset demonstrated a significant correlation for both datasets, where a decrease in rank score was associated with disease state (Figures 5(e) and 6(e)).

## 4. Discussion

A decrease in* SPRY* expression has been reported in cancers including those of the prostate [25, 26] and breast [19]. Their role in antagonising receptor tyrosine kinase signalling and an expanding list of tumours in which they are apparently downregulated has led to them being considered as tumour suppressors. Further evidence of their role as important tumour suppressors comes from a recent study of* Spry1* null mice thyroids which demonstrated that Sprouty can act, independently of the ERK pathway, in hyperproliferative cells to induce senescence via NF*κ*B signalling [34].

The results presented here provide further evidence to support Sprouty as a tumour suppressor. Surprisingly, we have demonstrated that single germline deletions of either* Spry1* or* Spry2* result in the development of prostatic intraepithelial neoplasias, the generally accepted precursor of prostate cancer [35]. Moreover, this was associated with significant increases in proliferative cells, as determined by PCNA analysis in both hemi- and homozygous Spry null mice. These findings were contrary to our original hypothesis that single gene deletions would not result in significant pathologies, based on the study of Schutzman and Martin [27]. It is important to note that in that study a prostate-specific deletion of both* Spry1* and* Spry2* induced LGPIN only. This is in stark contrast to our study where hemizygous mice had HGPIN. It is unclear as to why this difference, and we cannot discount off target effects in our germline deletions that affect other cell types that are important in the development of prostate cancer, such as neuroendocrine cells or stromal cells. These cell types are important in the development of prostate cancer, not least the reactive stroma. A feature of reactive stroma is the induced myodifferentiation of fibroblasts associated with TGF-*β* [36]. Deregulation of TGF-*β* signalling in the stroma is known to be associated with prostate cancer development. Suppression of TGF-*β* signalling in mouse prostatic stroma has been shown to induce PIN formation, whilst hyperstimulation of stromal cells by TGF-*β* induces tumorigenesis [37]. Both Spry1 and Spry2 have recently been shown to be negative regulators of TGF-*β* signalling [38]. It is likely then that reduced sprouty in stroma may result in increased TGF-*β* signalling. As TGF-*β* stimulates bFGF secretion by stroma [39], we suggest that in our model there is a compound effect of greater FGF present, with decreased attenuation of signalling, resulting in the formation of PIN in both* Spry1* ^+/−^ and* Spry2* ^+/−^ prostates.

Whilst prostates of* Spry1* ^−/−^ mice had a similar total PIN incidence as* Spry1* ^+/−^ mice, they displayed a significantly lower incidence of HGPIN. This suggests a degree of redundancy in the sprouty tumour suppressors, with Spry2 possibly being compensatory in this context. This might also explain why PIN pathology was not seen in one of the* Spry1* ^−/−^ mice. As all* Spry1* ^+/−^ mice assessed had PIN pathology, it is possible that a dose effect is seen such that* Spry2 *is increased in* Spry1* null mice to compensate where* Spry2* is the most important of the sprouty family negative regulators of FGF signalling. That all* Spry2* ^+/−^ and* Spry2* ^−/−^ prostates assessed displayed PIN pathology with* Spry2* ^−/−^ mice prostates having the greatest total PIN incidence, the highest levels of HGPIN, and the most proliferative epithelial cells than all other mice studied supports this. Indeed, concomitant loss of Spry1 and Spry2 function results in tumorigenesis [27] with significant PIN and invasive tumours only induced by codeletion of* Spry1 *and* Spry2* in haploinsufficient phosphatase and tensin (Pten) mice. Significantly,* Pten* null mice develop prostate cancer [40]. PTEN activity is necessary for the activity of SPRY2 in HeLa cells where silencing PTEN diminished SPRY2-mediated inhibition of cell proliferation [41]. Overexpression of* SPRY2* increased total PTEN and increased the amount of the more active dephosphorylated PTEN [41]. Such crosstalk between cytokine signalling pathways and evidence for sprouty suppression/activation of urokinase and NF*κ*B [35] is a classic example of redundancy in regulation of signalling pathways.

Another possibility is that more specific inhibitors of the ERK/MAPK pathway are involved. One family that could provide this role is the sprouty-related (SPRED) family of proteins. We investigated this further in a preliminary study of publicly available datasets of human prostate cancer. Consistent with our hypothesis, in both datasets analysed, a significant cosuppression of SPRYs and SPREDs is evident. This is the first description of such cosuppression of the Sprouty and Sprouty-related negative regulators to our knowledge. Only one report exists with regard to SPREDs in prostate cancer, describing evidence for a loss of* SPRED2* expression in high Gleason grade lesions [28]. Similarly, in both datasets assessed here,* SPRED2* expression is significantly suppressed in prostate cancer tissues. However, this is only evident in primary tissues, with no further decrease in expression evident in metastatic cancers.* SPRED1 *expression whilst suppressed in one dataset did not show any significant change in another. Hence, no clear conclusion with regard to its role in prostate cancer development can be drawn, and this warrants further extensive study in the clinical setting. Of note is our finding that* SPRY1* expression does not appear to be significantly reduced in prostate carcinomas. This is in contrast to a previous study that suggested there was a reduction in* SPRY1* at the gene level, albeit in a smaller sample size, where 16 of 20 tissue samples showed reduced mRNA compared to the normal [25]. Our description of reduced* SPRY2* expression is consistent with other studies of clinical samples of prostate cancer [25, 26].

In conclusion, loss of a single allele of either* Spry1* or* Spry2* results in the development of prostate intraepithelial neoplasia in mice. These findings demonstrate the importance of negative regulators of receptor tyrosine signalling, such as Spry, in the clinical setting. Our observation that there is a concomitant loss of* SPRY 2* with* SPREDs 1* and* 2* in human prostate cancers supports this hypothesis and suggests that a loss of both Sprouty and Spreds is important in prostate cancer development. As such, these negative regulators of receptor tyrosine kinase signalling provide interesting targets for future pharmacopeia.
